# SVM clustering

**DOI:** 10.1186/1471-2105-8-S7-S18

**Published:** 2007-11-01

**Authors:** Stephen Winters-Hilt, Sam Merat

**Affiliations:** 1 Department of Computer Science, University of New Orleans, LA, 70148, USA; 2 Research Institute for Children, Children's Hospital, New Orleans, LA, 70118, USA

## Abstract

**Background:**

Support Vector Machines (SVMs) provide a powerful method for classification (supervised learning). Use of SVMs for clustering (unsupervised learning) is now being considered in a number of different ways.

**Results:**

An SVM-based clustering algorithm is introduced that clusters data with no *a priori *knowledge of input classes. The algorithm initializes by first running a binary SVM classifier against a data set with each vector in the set randomly labelled, this is repeated until an *initial convergence *occurs. Once this *initialization *step is complete, the SVM confidence parameters for classification on each of the training instances can be accessed. The lowest confidence data (e.g., the worst of the mislabelled data) then has its' labels switched to the other class label. The SVM is then re-run on the data set (with partly re-labelled data) and is guaranteed to converge in this situation since it converged previously, and now it has fewer data points to carry with mislabelling penalties. This approach appears to limit exposure to the local minima traps that can occur with other approaches. Thus, the algorithm then improves on its weakly convergent result by SVM re-training after each re-labeling on the worst of the misclassified vectors – i.e., those feature vectors with confidence factor values beyond some threshold. The repetition of the above process improves the accuracy, here a measure of separability, until there are no misclassifications. Variations on this type of clustering approach are shown.

**Conclusion:**

Non-parametric SVM-based clustering methods may allow for much improved performance over parametric approaches, particularly if they can be designed to inherit the strengths of their supervised SVM counterparts.

## Introduction

### Support Vector Machine

**Figure 1:**
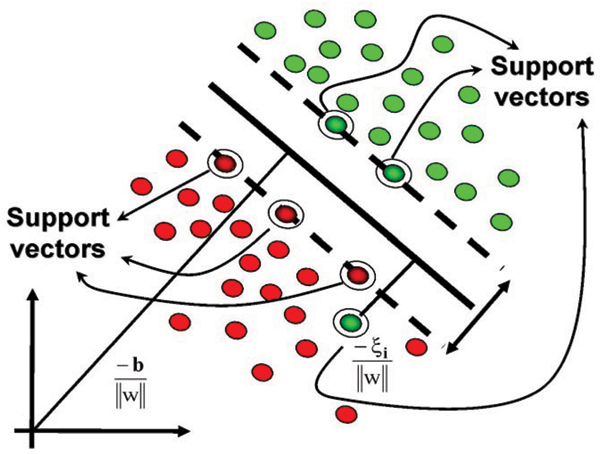
Support Vector Machine uses Structural Risk Minimization to compare various separation models and to eventually choose the model with the largest margin of separation.

Support Vector Machines (SVMs) provide a very efficient mechanism to construct a separating hyperplane (see Fig. 1), surrounded by the thickest margin, using a set of training data. Prediction is made according to some measure of the distance between the test data and the hyperplane. Cover's theorem states that complex pattern-classification problems can be transformed into a new feature space where the patterns are more likely to be linearly separable, provided that the transformation itself is nonlinear and that the feature space is in a high enough dimension [1]. The SVM method achieves such a transformation by choosing a qualified kernel.

The SVM is briefly reviewed here using the notation of [2], more discussion is available there on implementation issues. Feature vectors are denoted by x_ik_, where index i labels the M feature vectors (1 ≤ i ≤ M) and index k labels the N feature vector components (1 ≤ i ≤ N). For the binary SVM, labeling of training data is done using label variable y_i _= ± 1 (with sign according to whether the training instance was from the positive or negative class). For hyperplane separability, elements of the training set must satisfy the following conditions: w_*β*_x_i*β*_-b ≥ +1 for i such that y_i _= +1, and w_*β*_x_i*β*_-b ≤ -1 for y_i _= -1, for some values of the coefficients w_1_,..., w_N_, and b (using the convention of implied sum on repeated Greek indices). This can be written more concisely as: y_i_(w_*β*_x_i*β*_-b) -1 ≥ 0. Data points that satisfy the equality in the above are known as "support vectors" (or "active constraints").

Once training is complete, discrimination is based solely on position relative to the discriminating hyperplane: w_*β*_x_i*β *_- b = 0. The boundary hyperplanes on the two classes of data are separated by a distance 2/w, known as the "margin," where w^2 ^= w_*β*_w_*β*_. By increasing the margin between the separated data as much as possible the SVM's optimal separating hyperplane is obtained. In the usual SVM formulation, the goal to maximize w^-1 ^is restated as the goal to minimize w^2^. The Lagrangian variational formulation then selects an optimum defined at a saddle point of L(w, b; *α*) = (w_*β*_w_*β*_)/2 - *α*_*γ *_y_*γ *_(w_*β*_x_*γβ*_-b) - *α*, where *α *= ∑_*γ*_*α*_*γ*_, *α*_*γ *_≥ 0 (1 ≤ *γ *≤ M). The saddle point is obtained by minimizing with respect to {w_1_,..., w_N_, b} and maximizing with respect to {*α*_1_,..., *α*_M_}. Further details are left to [2]. A Wolfe transformation is performed on the Lagrangian [2], and it is found that the training data (support vectors in particular, KKT class (ii) above) enter into the Lagrangian solely via the inner product x_i*β*_x_j*β*_. Likewise, the discriminator f_i_, and KKT relations, are also dependent on the data solely via the x_i*β*_x_j*β *_inner product. Generalization of the SVM formulation to data-dependent inner products other than x_i*β*_x_j*β *_are possible and are usually formulated in terms of the family of symmetric positive definite functions (reproducing kernels) satisfying Mercer's conditions [3,4].

### SVM-internal clustering

Clustering, the problem of grouping *objects *based on their known similarities is studied in various publications [2,5,7]. SVM-*Internal *Clustering [2,7] (our terminology, usually referred to as a one-class SVM) uses internal aspects of Support Vector Machine formulation to find the smallest enclosing sphere. Let {*x*_*i*_} be a data set of *N *points in *R*^*d *^(*Input Space*.) Similar to the nonlinear SVM formulation, using a non-linear transformation *φ*, we transform *x *to a high-dimensional space – *Kernel space *– and look for the smallest enclosing sphere of radius *R*. Hence we have:

||*φ*(*x*_*j*_) - *a *||^2 ^≤ *R*^2 ^for all *j *= 1,..., *N*

where *a *is the center of the sphere. Soft constraints are incorporated by adding slack variables *ζ*_j_:

‖ϕ(xj)−a‖2≤R2+ζjforall j=1,...,NSubject to:ζj≥0
 MathType@MTEF@5@5@+=feaafiart1ev1aaatCvAUfKttLearuWrP9MDH5MBPbIqV92AaeXatLxBI9gBaebbnrfifHhDYfgasaacH8akY=wiFfYdH8Gipec8Eeeu0xXdbba9frFj0=OqFfea0dXdd9vqai=hGuQ8kuc9pgc9s8qqaq=dirpe0xb9q8qiLsFr0=vr0=vr0dc8meaabaqaciaacaGaaeqabaqabeGadaaakeaafaqabeGabaaabaqbaeqabeGaaaqaamaafmaabaacciGae8x1dOMaeiikaGIaemiEaG3aaSbaaSqaaiabdQgaQbqabaGccqGGPaqkcqGHsislcqWGHbqyaiaawMa7caGLkWoadaahaaWcbeqaaiabikdaYaaakiabgsMiJkabdkfasnaaCaaaleqabaGaeGOmaidaaOGaey4kaSIae8NTdO3aaSbaaSqaaGqaciab+PgaQbqabaaakeaaieaacqqFMbGzcqqFVbWBcqqFYbGCcqqFGaaicqqFHbqycqqFSbaBcqqFSbaBcqqGGaaicqWGQbGAcqGH9aqpcqaIXaqmcqGGSaalcqGGUaGlcqGGUaGlcqGGUaGlcqGGSaalcqWGobGtaaaabaGae03uamLae0xDauNae0NyaiMae0NAaOMae0xzauMae03yamMae0hDaqNaeeiiaaIae0hDaqNae03Ba8MaeiOoaOJae8NTdO3aaSbaaSqaaiab+PgaQbqabaGccqGHLjYScqaIWaamaaaaaa@6864@

We formulate the Lagrangian as:

L=R2−∑jβj(R2+ζj−‖ϕ(xj)−a‖2)−∑jζjμj+C∑jζjsubject to:βj≥0,μj≥0,
 MathType@MTEF@5@5@+=feaafiart1ev1aaatCvAUfKttLearuWrP9MDH5MBPbIqV92AaeXatLxBI9gBaebbnrfifHhDYfgasaacH8akY=wiFfYdH8Gipec8Eeeu0xXdbba9frFj0=OqFfea0dXdd9vqai=hGuQ8kuc9pgc9s8qqaq=dirpe0xb9q8qiLsFr0=vr0=vr0dc8meaabaqaciaacaGaaeqabaqabeGadaaakeaafaqabeGabaaabaGaemitaWKaeyypa0JaemOuai1aaWbaaSqabeaacqaIYaGmaaGccqGHsisldaaeqaqaaGGaciab=j7aInaaBaaaleaacqWGQbGAaeqaaOGaeiikaGIaemOuai1aaWbaaSqabeaacqaIYaGmaaGccqGHRaWkcqWF2oGEdaWgaaWcbaGaemOAaOgabeaakiabgkHiTmaafmaabaGae8x1dOMaeiikaGIaemiEaG3aaSbaaSqaaiabdQgaQbqabaGccqGGPaqkcqGHsislcqWGHbqyaiaawMa7caGLkWoadaahaaWcbeqaaiabikdaYaaakiabcMcaPiabgkHiTaWcbaGaemOAaOgabeqdcqGHris5aOWaaabeaeaacqWF2oGEdaWgaaWcbaGaemOAaOgabeaakiab=X7aTnaaBaaaleaacqWGQbGAaeqaaOGaey4kaSIaem4qam0aaabeaeaacqWF2oGEdaWgaaWcbaGaemOAaOgabeaaaeaacqWGQbGAaeqaniabggHiLdaaleaacqWGQbGAaeqaniabggHiLdaakeaaieaacqGFZbWCcqGF1bqDcqGFIbGycqGFQbGAcqGFLbqzcqGFJbWycqGF0baDcqqGGaaicqGF0baDcqGFVbWBcqGG6aGocqWFYoGydaWgaaWcbaGaemOAaOgabeaakiabgwMiZkabicdaWiabcYcaSiab=X7aTnaaBaaaleaacqWGQbGAaeqaaOGaeyyzImRaeGimaaJaeiilaWcaaaaa@7DB5@

where *C *is the cost for outliers and therefore *C*∑_*j*_*ζ*_*j *_is the penalty term. Taking the derivative of *L *w.r.t. *R*, *a *and *ζ *and setting them to zero we have:

∑jβj=1,a=∑jβjϕ(xj), andβj=C−μj.
 MathType@MTEF@5@5@+=feaafiart1ev1aaatCvAUfKttLearuWrP9MDH5MBPbIqV92AaeXatLxBI9gBaebbnrfifHhDYfgasaacH8akY=wiFfYdH8Gipec8Eeeu0xXdbba9frFj0=OqFfea0dXdd9vqai=hGuQ8kuc9pgc9s8qqaq=dirpe0xb9q8qiLsFr0=vr0=vr0dc8meaabaqaciaacaGaaeqabaqabeGadaaakeaafaqabeWabaaabaWaaabeaeaaiiGacqWFYoGydaWgaaWcbaGaemOAaOgabeaaaeaacqWGQbGAaeqaniabggHiLdGccqGH9aqpcqaIXaqmcqGGSaalaeaacqWGHbqycqGH9aqpdaaeqaqaaiab=j7aInaaBaaaleaacqWGQbGAaeqaaaqaaiabdQgaQbqab0GaeyyeIuoakiab=v9aQjabcIcaOiabdIha4naaBaaaleaacqWGQbGAaeqaaOGaeiykaKIaeiilaWIaeeiiaaccbaGae4xyaeMae4NBa4Mae4hzaqgabaGae8NSdi2aaSbaaSqaaiabdQgaQbqabaGccqGH9aqpcqWGdbWqcqGHsislcqWF8oqBdaWgaaWcbaGaemOAaOgabeaakiabc6caUaaaaaa@5536@

Substituting the above equations back into the Lagrangian, we have the following dual formalism:

W=1−∑i,jβiβjKijwhere 0≤βi≤C;Kij=exp(−‖xi−xj‖2/2σ2)subject to:∑iβi=1
 MathType@MTEF@5@5@+=feaafiart1ev1aaatCvAUfKttLearuWrP9MDH5MBPbIqV92AaeXatLxBI9gBaebbnrfifHhDYfgasaacH8akY=wiFfYdH8Gipec8Eeeu0xXdbba9frFj0=OqFfea0dXdd9vqai=hGuQ8kuc9pgc9s8qqaq=dirpe0xb9q8qiLsFr0=vr0=vr0dc8meaabaqaciaacaGaaeqabaqabeGadaaakeaafaqabeGabaaabaqbaeqabeWaaaqaaiabdEfaxjabg2da9iabigdaXiabgkHiTmaaqababaacciGae8NSdi2aaSbaaSqaaiabdMgaPbqabaGccqWFYoGydaWgaaWcbaGaemOAaOgabeaakiabdUealnaaBaaaleaacqWGPbqAcqWGQbGAaeqaaaqaaiabdMgaPjabcYcaSiabdQgaQbqab0GaeyyeIuoaaOqaaiabbEha3jabbIgaOjabbwgaLjabbkhaYjabbwgaLjabbccaGiabicdaWiabgsMiJkab=j7aInaaBaaaleaacqWGPbqAaeqaaOGaeyizImQaem4qamKaei4oaSdabaGaem4saS0aaSbaaSqaaiabdMgaPjabdQgaQbqabaGccqGH9aqpieGacqGFLbqzcqGF4baEcqGFWbaCcqGGOaakcqGHsisldaqbdaqaaiabdIha4naaBaaaleaacqWGPbqAaeqaaOGaeyOeI0IaemiEaG3aaSbaaSqaaiabdQgaQbqabaaakiaawMa7caGLkWoadaahaaWcbeqaaiabikdaYaaakiabc+caViabikdaYiab=n8aZnaaCaaaleqabaGaeGOmaidaaOGaeiykaKcaaaqaaGqaaiab9nhaZjab9vha1jab9jgaIjab9PgaQjab9vgaLjab9ngaJjab9rha0jabbccaGiab9rha0jab99gaVjabcQda6maaqababaGae8NSdi2aaSbaaSqaaiabdMgaPbqabaGccqGH9aqpcqaIXaqmaSqaaiabdMgaPbqab0GaeyyeIuoaaaaaaa@845C@

By KKT relations we have:

*ζ*_*j*_*μ*_*j *_= 0 and *β*_*j*_(*R*^2 ^+ *ζ*_*j *_- ||*φ*(*x*_*j*_) - *a *||^2^) = 0.

In the *feature space*, *β*_*j *_= *C *only if *ζ*_*j *_> 0; hence it lies outside of the sphere i.e. *R*^2 ^< ||*φ*(*x*_*j*_) - *a *||^2^. This point becomes a bounded support vector or BSV. Similarly if *ζ*_*j *_= 0, and 0 <*β*_*j *_< C, then it lies on the surface of the sphere i.e. *R*^2 ^= ||*φ*(*x*_*j*_) - *a *||^2^. This point becomes a support vector or SV. If *ζ*_*j *_= 0, and *β*_*j *_= 0, then *R*^2 ^> ||*φ*(*x*_*j*_) - *a *||^2 ^and hence this point is enclosed with-in the sphere.

### SVM-external clustering

Although the internal approach to SVM clustering is only weakly biased towards the shape of the clusters in *feature *space (the bias is for spherical clusters in the *kernel *space), it still lacks robustness. In the case of most real-world problems and strongly overlapping clusters, the SVM-Internal Clustering algorithm above can only delineate the relatively small cluster cores. Additionally, the implementation of the formulation is tightly coupled with the initial choice of kernel; hence the static nature of the formulation and implementation does not accommodate numerous kernel tests. To remedy this excessive geometric constraint, an external-SVM clustering algorithm is introduced in [2] that clusters data vectors with no a priori knowledge of each vector's class.

The algorithm works by first running a Binary SVM against a data set, with each vector in the set randomly labeled, until the SVM converges. In order to obtain convergence, an acceptable number of KKT violators must be found. This is done through running the SVM on the randomly labeled data with different numbers of allowed violators until the number of violators allowed is near the lower bound of violators needed for the SVM to converge on the particular data set. Choice of an appropriate kernel and an acceptable sigma value also will affect convergence. After the initial convergence is achieved, the (sensitivity + specificity) will be low, likely near 1. The algorithm now improves this result by iteratively re-labeling *only *the worst misclassified vectors, which have confidence factor values beyond some threshold, followed by rerunning the SVM on the newly relabeled data set. This continues until no more progress can be made. Progress is determined by an increasing value of (sensitivity+specificity), hopefully nearly reaching 2. This method provides a way to cluster data sets without prior knowledge of the data's clustering characteristics, or the number of clusters. In practice, the initialization step, that arrives at the first SVM convergence, typically takes longer than all subsequent partial re-labeling and SVM rerunning steps.

The SVM-External clustering approach is not biased towards the shape of the clusters, and unlike the internal approach the formulation is not fixed to a single kernel class. Nevertheless, there are robustness and consistency issues that arise in SVM-External clustering approache. To rectify this issue, an external approach to SVM clustering is prescribed herein, that takes into account the robustness required in realistic applications.

### Supervised cluster evaluation

Externally derived class labels require external categorization that assume "correct" labels for each category. Unlike classification, clustering algorithms do not have access to the same level of fundamental truth. Thus, the performance of unsupervised algorithms, such as clustering, can't be measured with the same certitude as for the classification problems. In this paper the result of the clustering is measured using the externally derived class labels for the patterns. Subsequently, we can use some of the classification-oriented measures to evaluate our results. These measures evaluate the extent to which a cluster contains patterns of a single class (see [4]).

The measures of prediction accuracy used here, Sensitivity, *SN*, and Specificity, *SP*, etc. are defined as follows:

*SN *= The fraction of positive patterns predicted correctly by the model = *TP*/(*TP *+ *FN*)

*SP *= The fraction of predicted patterns that turns out to be positive = *TP*/(*TP *+ *FP*)

*nSN *= The fraction of negative patterns predicted correctly by the model = *TN*/(*TN *+ *FP*)

*nSP *= The fraction of predicted patterns that turns out to be negative = *TN*/(*TN *+ *FN*)

The two measures used in this paper are Entropy and Purity, and are based on the pair {SP, nSP}, see Discussion for further details.

### Cluster entropy and purity

Let *p*_*ij *_be the probability that an object in cluster *i *belongs to class *j*. Then entropy for the cluster *i*, e_i_, can be written as:

ei=−∑j=1Jpijlog⁡pij
 MathType@MTEF@5@5@+=feaafiart1ev1aaatCvAUfKttLearuWrP9MDH5MBPbIqV92AaeXatLxBI9gBaebbnrfifHhDYfgasaacH8akY=wiFfYdH8Gipec8Eeeu0xXdbba9frFj0=OqFfea0dXdd9vqai=hGuQ8kuc9pgc9s8qqaq=dirpe0xb9q8qiLsFr0=vr0=vr0dc8meaabaqaciaacaGaaeqabaqabeGadaaakeaaieaacqWFLbqzdaWgaaWcbaGae8xAaKgabeaakiabg2da9iabgkHiTmaaqahabaGaemiCaa3aaSbaaSqaaiabdMgaPjabdQgaQbqabaGccyGGSbaBcqGGVbWBcqGGNbWzcqWGWbaCdaWgaaWcbaGaemyAaKMaemOAaOgabeaaaeaaieGacqGFQbGAcqGH9aqpcqaIXaqmaeaaieqacqqFkbGsa0GaeyyeIuoaaaa@44F7@

where J is the number of classes. Similarly, the purity for the cluster *i*, *p*_*i*_, can be expressed as,

pi=max⁡jpij
 MathType@MTEF@5@5@+=feaafiart1ev1aaatCvAUfKttLearuWrP9MDH5MBPbIqV92AaeXatLxBI9gBaebbnrfifHhDYfgasaacH8akY=wiFfYdH8Gipec8Eeeu0xXdbba9frFj0=OqFfea0dXdd9vqai=hGuQ8kuc9pgc9s8qqaq=dirpe0xb9q8qiLsFr0=vr0=vr0dc8meaabaqaciaacaGaaeqabaqabeGadaaakeaacqWGWbaCdaWgaaWcbaGaemyAaKgabeaakiabg2da9maaxababaGagiyBa0MaeiyyaeMaeiiEaGhaleaacqWGQbGAaeqaaOGaemiCaa3aaSbaaSqaaiabdMgaPjabdQgaQbqabaaaaa@3ABF@

Note that the probability that an object in cluster *i *belongs to class *j *can be written as the number of objects of class *j *in cluster *i*, *n*_*ij*_, divided by the total number of objects in cluster *i*, *n*_*i*_, (*i.e.*, *p*_*ij *_= *n*_*ij*_/*n*_*i*_) Using this notation the overall validity of a cluster *i *using the measure *f*_*i *_(for either entropy or purity) is the weighted sum of that measure over all clusters. Hence,

fi=1/N∑i=1Knifi
 MathType@MTEF@5@5@+=feaafiart1ev1aaatCvAUfKttLearuWrP9MDH5MBPbIqV92AaeXatLxBI9gBaebbnrfifHhDYfgasaacH8akY=wiFfYdH8Gipec8Eeeu0xXdbba9frFj0=OqFfea0dXdd9vqai=hGuQ8kuc9pgc9s8qqaq=dirpe0xb9q8qiLsFr0=vr0=vr0dc8meaabaqaciaacaGaaeqabaqabeGadaaakeaacqWGMbGzdaWgaaWcbaGaemyAaKgabeaakiabg2da9iabigdaXiabc+caVGqaaiab=5eaonaaqahabaGae8NBa42aaSbaaSqaaiab=LgaPbqabaGccqWFMbGzdaWgaaWcbaGae8xAaKgabeaaaeaacqWGPbqAcqGH9aqpcqWFXaqmaeaacqWFlbWsa0GaeyyeIuoaaaa@3FF4@

where, *K *is number of clusters and *N *is the total number of patterns. For our 2-class clustering problem:

*p*1 = *max*(*SP*, 1 - *SP*), *p*2 = *max*(*nSP*, 1 - *nSP*)

and:

*purity *= *max*((*TP *+ *TN*)/(*TP *+ *FP *+ *TN *+ *FN*); 1-(*TP *+ *TN*)/(*TP *+ *FP *+ *TN *+ *FN*))

Although similar, *entropy *is a more comprehensive measure than *purity*. Rather than considering either the frequency of patterns that are within a class or the frequency of patterns that are outside of a class, *entropy*, takes into account the entire distribution.

### Unsupervised cluster evaluation

Unsupervised evaluation techniques do not depend on external class information. These measures are often optimization functions in many clustering algorithms. Sum-of-Squared-Error (SSE) measures the compactness of a single cluster and other measures evaluate the isolation of a cluster from other clusters.

Sum-of-Squared-Error. SSE, in input space, can be written as:

Je=12∑i=1Knis^i
 MathType@MTEF@5@5@+=feaafiart1ev1aaatCvAUfKttLearuWrP9MDH5MBPbIqV92AaeXatLxBI9gBaebbnrfifHhDYfgasaacH8akY=wiFfYdH8Gipec8Eeeu0xXdbba9frFj0=OqFfea0dXdd9vqai=hGuQ8kuc9pgc9s8qqaq=dirpe0xb9q8qiLsFr0=vr0=vr0dc8meaabaqaciaacaGaaeqabaqabeGadaaakeaaieaacqWFkbGsdaWgaaWcbaGae8xzaugabeaakiabg2da9maaliaabaGaeGymaedabaGaeGOmaidaamaaqahabaGae8NBa42aaSbaaSqaaiab=LgaPbqabaGccuWFZbWCgaqcamaaBaaaleaacqWFPbqAaeqaaaqaaiab=LgaPjabg2da9iabigdaXaqaaiab=TealbqdcqGHris5aaaa@3ED8@

where for any similarity function *s*(x, x')

s^i=1/ni2∑x∈Di∑x∈Dis(x,x′)
 MathType@MTEF@5@5@+=feaafiart1ev1aaatCvAUfKttLearuWrP9MDH5MBPbIqV92AaeXatLxBI9gBamXvP5wqSXMqHnxAJn0BKvguHDwzZbqegyvzYrwyUfgarqqtubsr4rNCHbGeaGqiA8vkIkVAFgIELiFeLkFeLk=iY=Hhbbf9v8qqaqFr0xc9pk0xbba9q8WqFfeaY=biLkVcLq=JHqVepeea0=as0db9vqpepesP0xe9Fve9Fve9GapdbaqaaeGacaGaaiaabeqaamqadiabaaGcbaacdaGab83CayaajaWaaSbaaSqaaiaa=LgaaeqaaOGaeyypa0JaeGymaeJaei4la8Iaa8NBamaaDaaaleaacaWFPbaabaGaeGOmaidaaOWaaabuaeaadaaeqbqaaiabdohaZjabcIcaOiaa=HhacqGGSaalceWF4bGbauaacqGGPaqkaSqaaiaa=HhacqGHiiIZcaWFebWaaSbaaWqaaiaa=LgaaeqaaaWcbeqdcqGHris5aaWcbaGaa8hEaiabgIGiolaa=readaWgaaadbaGaa8xAaaqabaaaleqaniabggHiLdaaaa@5805@

Due the arising mathematical complexity, it is often convenient to use Euclidean distance as the measure of similarity. Hence,

*s*(x, x') = ||x - x'||^2^

Let *φ*: *X *→ F and *k*(x, y) = {*φ *(x), *φ *(y)}, then J_e _can be rewritten (this time feature space) as:

Je=∑i=1KJi
 MathType@MTEF@5@5@+=feaafiart1ev1aaatCvAUfKttLearuWrP9MDH5MBPbIqV92AaeXatLxBI9gBaebbnrfifHhDYfgasaacH8akY=wiFfYdH8Gipec8Eeeu0xXdbba9frFj0=OqFfea0dXdd9vqai=hGuQ8kuc9pgc9s8qqaq=dirpe0xb9q8qiLsFr0=vr0=vr0dc8meaabaqaciaacaGaaeqabaqabeGadaaakeaaieaacqWFkbGsdaWgaaWcbaGae8xzaugabeaakiabg2da9maaqahabaGae8NsaO0aaSbaaSqaaiab=LgaPbqabaaabaGae8xAaKMaeyypa0JaeGymaedabaGae83saSeaniabggHiLdaaaa@3994@

for

Ji=∑x∈Dik(x,x)−1/ni∑x∈Di∑x′∈Dik(x,x′)
 MathType@MTEF@5@5@+=feaafiart1ev1aaatCvAUfKttLearuWrP9MDH5MBPbIqV92AaeXatLxBI9gBaebbnrfifHhDYfgasaacH8akY=wiFfYdH8Gipec8Eeeu0xXdbba9frFj0=OqFfea0dXdd9vqai=hGuQ8kuc9pgc9s8qqaq=dirpe0xb9q8qiLsFr0=vr0=vr0dc8meaabaqaciaacaGaaeqabaqabeGadaaakeaaieaacqWFkbGsdaWgaaWcbaGae8xAaKgabeaakiabg2da9maaqafabaGae83AaSMaeiikaGIae8hEaGNaeiilaWIae8hEaGNaeiykaKIaeyOeI0IaeGymaeJaei4la8Iae8NBa42aaSbaaSqaaiab=LgaPbqabaGcdaaeqbqaamaaqafabaGae83AaSMaeiikaGIae8hEaGNaeiilaWIaf8hEaGNbauaacqGGPaqkaSqaaiqb=Hha4zaafaGaeyicI4Sae8hraq0aaSbaaWqaaiab=LgaPbqabaaaleqaniabggHiLdaaleaacqWF4baEcqGHiiIZcqWFebardaWgaaadbaGae8xAaKgabeaaaSqab0GaeyyeIuoaaSqaaiab=Hha4jabgIGiolab=reaenaaBaaameaacqWFPbqAaeqaaaWcbeqdcqGHris5aaaa@5B00@

Note that SSE, like any other unsupervised criterion, may not reveal the true underlying clusters, since the Euclidean distance simplification favors spherically shaped clusters. However, this geometry is imposed after the data was mapped to the feature space.

## Results

### Re-labeler

**Figure 2:**
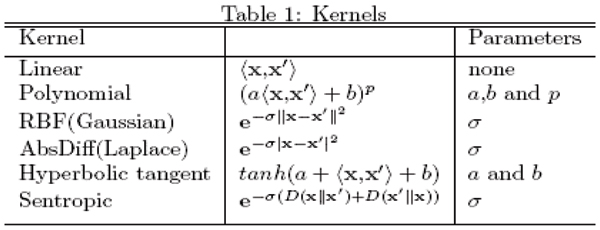
The choice of kernel along with a genuine set of kernel parameters is important as the above table summarizes some of the most popular kernels used for classification and clustering.

**Figure 3:**
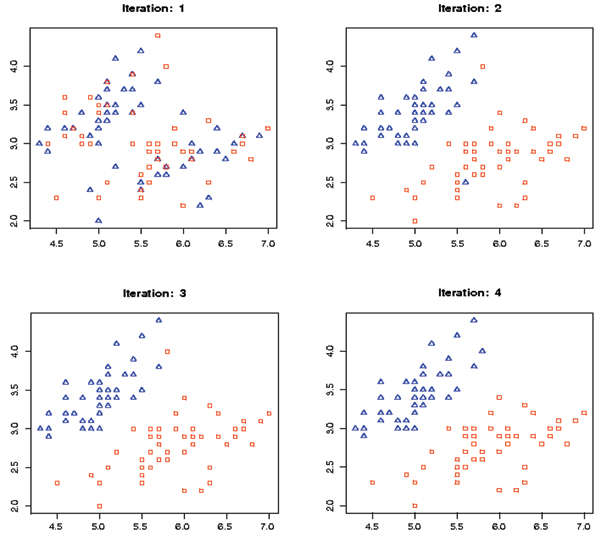
The results of SVM-Relabeler algorithm using the linear kernel.

**Figure 4:**
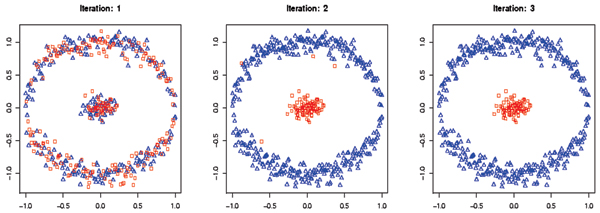
The results of SVM-Relabeler algorithm using a third degree polynomial kernel.

The geometry of the hyperplane depends on the kernel and the kernel parameters (see Figure 2). In Fig. 3, the decision hyperplane is linear in the feature space, while in Fig. 4 the decision hyperplane is circular in the feature space. The clustering kernel used in Fig. 3 was the linear kernel. The clustering kernel used in Fig. 4 was the polynomial kernel (the linear kernel failed in this case).

**Figure 5:**
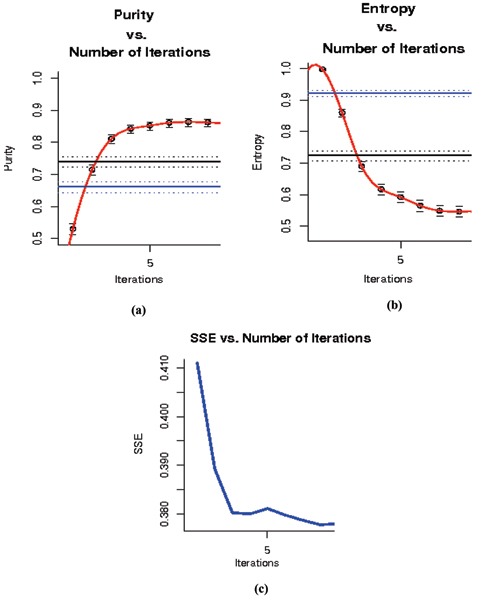
(a) and (b) show the boost in Purity and Entropy as a function of Number of Iterations after the completion the completion of the Re-labeler algorithm. (c) demonstrates SSE as an unsupervised evaluation mechanism that mimics purity and entropy as the measure of true clusters. The blue and black lines are the result of running fuzzy c-mean and kernel k-mean on the same dataset.

In the following experiments clustering is performed on a data set consisting of 8GC and 9GC DNA hairpin data (part of data sets used in [2]). The set consists of 400 elements. Half of the elements belong to each class. Although convergence is always achieved, convergence to a *global *optimum is not guaranteed. Figures 5a and 5b demonstrate the boost in Purity and Entropy (with the RBF kernel) as a function of Number of Iterations, while Fig 5c demonstrates this boost as decrement in SSE as a function of Number of Iterations. Note that the stopping criteria for the algorithm is based on the **unsupervised **measure of SSE. Comparison to fuzzy c-means and kernel k-means is shown on the same dataset (the solid blue and black lines in Fig. 5a and 5b). The greatly improved performance of the SVM-External approach over conventional clustering methods, consistent with results found in prior work [2], strongly motivates further developments along these lines. This completes the Re-labeler as an unsupervised method for clustering, further specifics on the Method are left to that section.

### Perturbation, random relabeling and hybrid methods

**Figure 6:**
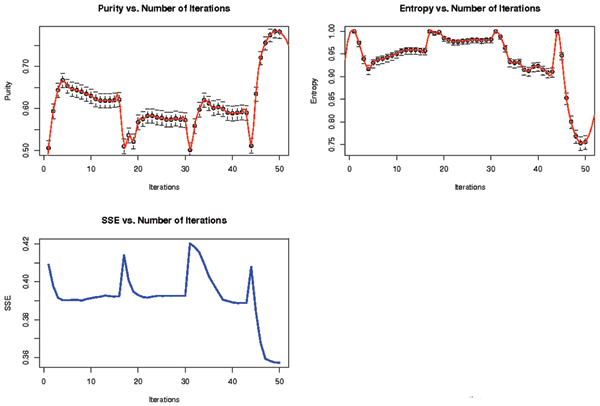
The result of Re-labeler Algorithm with Perturbation. The top plots demonstrate the various Purity and Entropy scores for each perturbed run. The spikes are drops followed by recovery in the validity of the clusters as a result of random perturbation. The bottom plot is a similar demonstration, by tracking the unsupervised quality of the clusters. Note that after 4 runs of perturbation best solution is recovered.

**Figure 7:**
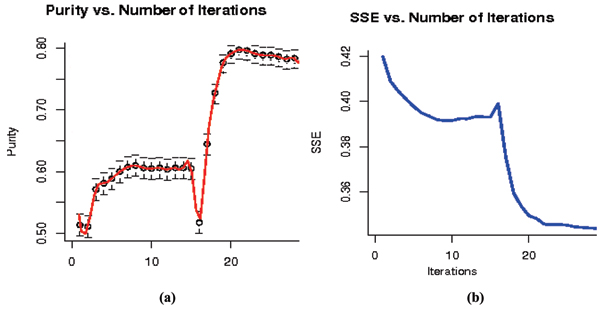
(a) and (b) represent the SSE and Purity evaluation of hybrid Re-labeler with Perturbation on the same dataset. Data is initially clustered using k-means to initialize the Re-labeler algorithm. The first segment of the plot (right before the spike at 16) is the result of Re-labeler after 10% perturbation, while the second segment is the result after 30% perturbation. Purity Number of Iterations.

It is found that the result of the Re-labeler algorithm can be significantly improved by randomly perturbing a weak clustering solution and repeating the SVM-external label-swapping iterations as depicted in Fig. 6. To explore this further, a hybrid SVM-external approach to the above problem is introduced to replace the initial random labeling step with k-means clustering or some other fast clustering algorithm. The initial SVM-external clustering must then be slightly and randomly perturbed to properly initialize the re-labeling step; otherwise the SVM clustering tends to return to the original k-means clustering solution. A complication is the unknown amount of perturbation of the k-means solution that is needed to initialize the SVM-clustering well – it is generally found that a weak clustering method does best for the initialization (or one weakened by a sufficient amount of perturbation). The results are shown in Figure 7. Note how the hybrid method can improve over k-means and SVM-clustering alone.

### SV-dropper

**Figure 8:**
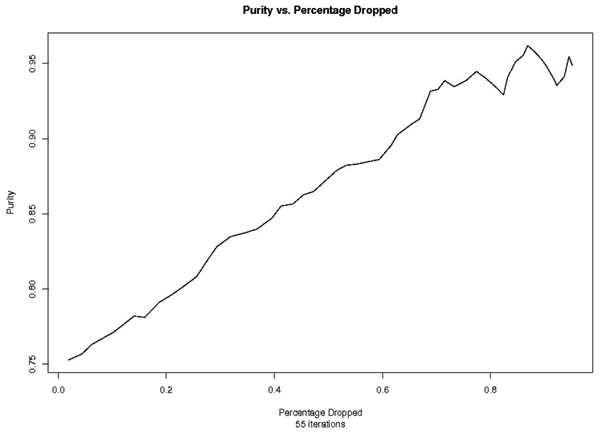
Purity vs Percentage Dropped after 55 iterations. At about 78% drop the data set becomes too small and the statistics begin to have much greater variance.

As explained in the Discussion section, identifying the hard-to-cluster data leads to the best overall performance. These weakly clustering data points have the greatest chance of being identified as noise, and therefore dropped out of the dataset. Results from running SV-dropper on the data are shown in Figure 8. The methods in the above results can be used together: the Hybrid Re-labeler can be run to get a solution, then backed off, using the SV dropper method, to establish highly accurate cluster "cores". These cores can then be used to seed the SVM-ABC algorithm, an area of ongoing work that is described in the Discussion section.

## Discussion

### Comparison to conventional clustering approaches

The proposed SVM-External approach to clustering appears to inherit the strengths of SVM classification – an amazing prospect. It is a non-parametric approach due to its manipulation of label assignment at the individual data instance level – thus there is not a clearly stated objective function (this is a strength). Solutions are global since they correspond to the final, global, solution of the SVM classification process. SVM-External solutions scale with the size of the dataset according to how an SVM-classifier would scale, multiplied by the number of iterations of the SVM (approximately 10, as a rough rule). Thus, clustering scales quadratically with the size of the data, but can be processed in chunks by the SVM according the SVMs nice distributable training properties. For clustering on multiple classes, the process would proceed along the lines of a multiclass discrimination solution with a binary SVM classifier – by iterative binary decomposition until sub-clusters can't be split (the stopping criterion for the decomposition). Comparison to established clustering methods are shown in Fig. 5 (see Results) and are more extensively explored in [4], where the overall strengths of the SVM-External clustering approach are clearly evident.

### Sum-of-Squares as a cluster evaluation

The fitness of the cluster identified by the SVM-external algorithm is modeled using the cohesion of the clusters. In other words, the fitness of a cluster can be tracked using the compactness of that cluster as the algorithm progresses. It is necessary to note that the notion of compactness as a way to evaluate clusters favours spherical clusters over clusters spread over a linear region. However, this limitation does not normally affect our methodology, since this limitation is imposed over the *kernel space*, and not the *input feature space*.

### Stopping criteria and convergence for Re-labeler algorithm

As depicted in Algorithm 1 below, Re-labeler consists of two main parts: *doSVM() *and *doRelabel() *functions. Since doRelabel() [Alg. 2] consists of sequential relabeling of a finite set of labels with a finite alphabet ({-1,+1}) the convergence of this function is always guaranteed. However, the convergence of *doSVM() *depends on the underlying SVM algorithm used. The particular SVM implementation used in Re-labeler Algorithm is Sequential Minimal Optimization, introduced by John C. Platt [8].

The basic stopping condition for the Re-labeler Algorithm is when there are no relabelings left to be performed. In terms of SSE, the unsupervised clustering measure, the algorithm halts when SSE remains unchanged. However, a decrease in SSE does not necessarily mean significant improvement to the quality of the clustering. Therefore, hypothesis testing and thresholding may prove useful depending on the application and the nature and geometry of the clustering data.

### SVM-ABC

New subtleties of classification-separation are possible with support vector machines via their direct handling and direct identification of data instances. Individual data points, in some instances, can be associated with "support vectors" at the boundaries between regions. By operating on labels of support vectors and focusing on training on certain subsets, the SVM-ABC algorithm offers the prospect to delineate highly complex geometries and graph-connectivity:

Split the clustering data into sets A, B and C

▪ A: Strong negatives

▪ C: Strong positives

▪ B: Weak negatives and weak positives

▪ Train an SVM on Data from A (labeled negative) and B (labeled positive)

▪ The support vectors: SV_AB

▪ Similarly train a new SVM on Data from C (labeled positive) and B (labeled negative)

▪ The support vectors: SV_CB

▪ Our objective is that the SV_AB and SV_CB sets have their labels flipped to be set A and C

▪ Regrow set A and C into the weak 'B' region.

▪ If an element of SV_AB is also in SV_BC, then the intersection of these sets are the elements that should be flipped to class B (if not already listed as class B).

▪ Stop at the first occurrence of any of these events

• Set B becomes empty

• Set B does not change

### Scoring binary classification conventions

In this paper we adopt the following conventions:

*SN *= *TP*/(*TP *+ *FN*)

*SP *= *TP*/(*TP *+ *FP*)

*nSN *= *TN*/(*TN *+ *FP*)

*nSP *= *TN*/(*TN *+ *FN*)

Bioinformatics researchers, in gene prediction for example [5], take as primary pair: {SN, SP}; ROC people, or people using a Confusion Matrix diagram, take as primary pair {SN, nSN}; Purity/Entropy researchers use {SP, nSP}; no one uses the pair {nSN, nSP} since it is a trivial label flip from being {SN, SP}. Label flipping leaves the sensitivity {SN, nSN} pair the same, similarly for the specificity pair {SP, nSP}. In other work we use the conventions that are commonly employed in gene prediction, and other instances where the signal identification is biased towards identification of positives. Specifically, they have two sets they focus on, the actual positives (genes) and the predicted positives (gene predictions). For gene prediction there is either a gene, or there is no-gene (i.e., junk, or background noise). In situations where your objective is to make the sets of actual positives (AP) and predicted positives (PP) maximally overlap, SP = specificity and SN = sensitivity are natural parameters and can be nicely described with a prediction accuracy Venn diagram (similar to a confusion matrix). For the problem here, we are adopting the purity/entropy measures to be in-line with other efforts in the clustering research community, so work with the pair {SP, nSP}.

### Rayleigh's criterion

Rayleigh's criterion, also known as the Rayleigh Limit, is used for the resolution of two light sources. In the case of two laser beam sources falling upon a single slit, the resolution limit is defined by the single-slit interference pattern where one source's maximum falls on the first minimum of the diffraction pattern of the second source. This definition is used for resolving distant stars as singletons or identification of binary star systems, etc. In the case of laser optics, the resolution of two sources can be pushed *beyond *the Rayleigh limit due to tracking the statistics of the individual photons that arrive. The relevance of all of this is that resolving two sources is equivalent to saying that a binary clustering solution exists, i.e., that the data is separable to some degree. If the clustering algorithm tracks the data instances individually, as it does with our SVM-external approach, we have a scenario analogous to the resolution in laser optics beyond the Rayleigh limit.

## Conclusion

A novel SVM-based clustering algorithm is explored that clusters data with no *a priori *knowledge of input classes. The resolution limit (i.e., clustering limit) of light sources, the classical Rayleigh Limit (see Discussion above), was eventually circumvented in laser-optics by probing the instance-based quantum-statistical description of quanta of light. With an SVM we have a non-parametric, instance-based, tracking as well. We hope to build upon the novel and powerful SVM clustering approach described here to eventually show clustering resolution beyond the "Parametric Limit", a limit that is otherwise imposed by the typical, parameterized, clustering methods, where the data isn't directly tracked but contributes to a parameterized model. Along these lines, it is hoped that further development of the SVM_ABC Algorithm described in the Discussion can offer recovery of subtle graph-like connectedness between cluster elements, a weakness of manifold-like separability approaches such as parametric-based clustering methods.

## Methods

### SVM-relabeler

The SVM classification formulation is used as the foundation for clustering a set of feature vectors with no *a priori *knowledge of the feature vector's classification. The non-separable SVM solution guarantees convergence at the cost of allowing misclassification. The extent of slack is controlled through the regularization constant, *C*, to penalize the slack variable, *ξ*. If the random mapping ((x_1_, y_1_),..., (x_m_, y_m_)) ∈ X^m ^× y is not linearly separable when ran through a binary SVM, the misclassified features are more likely to belong to the other cluster. Moreover, by relabeling those heavily misclassified features and by repeating this process we arrive at an optimal separation between the two clusters. The basics of this procedure is presented in Algorithm 1, where y^
 MathType@MTEF@5@5@+=feaafiart1ev1aaatCvAUfKttLearuWrP9MDH5MBPbIqV92AaeXatLxBI9gBaebbnrfifHhDYfgasaacH8akY=wiFfYdH8Gipec8Eeeu0xXdbba9frFj0=OqFfea0dXdd9vqai=hGuQ8kuc9pgc9s8qqaq=dirpe0xb9q8qiLsFr0=vr0=vr0dc8meaabaqaciaacaGaaeqabaqabeGadaaakeaacuWG5bqEgaqcaaaa@2E37@ is the new cluster assignment for x and *θ *contains *ω*, *α*, y'.

### Algorithm 1: SVM-Relabeler

Require: *m*, x

   1. y^
 MathType@MTEF@5@5@+=feaafiart1ev1aaatCvAUfKttLearuWrP9MDH5MBPbIqV92AaeXatLxBI9gBaebbnrfifHhDYfgasaacH8akY=wiFfYdH8Gipec8Eeeu0xXdbba9frFj0=OqFfea0dXdd9vqai=hGuQ8kuc9pgc9s8qqaq=dirpe0xb9q8qiLsFr0=vr0=vr0dc8meaabaqaciaacaGaaeqabaqabeGadaaakeaacuWG5bqEgaqcaaaa@2E37@ ← Randomly chosen from {-1,+1}

   2. repeat

   3. *θ *← *doSVM*(x, y^
 MathType@MTEF@5@5@+=feaafiart1ev1aaatCvAUfKttLearuWrP9MDH5MBPbIqV92AaeXatLxBI9gBaebbnrfifHhDYfgasaacH8akY=wiFfYdH8Gipec8Eeeu0xXdbba9frFj0=OqFfea0dXdd9vqai=hGuQ8kuc9pgc9s8qqaq=dirpe0xb9q8qiLsFr0=vr0=vr0dc8meaabaqaciaacaGaaeqabaqabeGadaaakeaacuWG5bqEgaqcaaaa@2E37@)

   4. y^
 MathType@MTEF@5@5@+=feaafiart1ev1aaatCvAUfKttLearuWrP9MDH5MBPbIqV92AaeXatLxBI9gBaebbnrfifHhDYfgasaacH8akY=wiFfYdH8Gipec8Eeeu0xXdbba9frFj0=OqFfea0dXdd9vqai=hGuQ8kuc9pgc9s8qqaq=dirpe0xb9q8qiLsFr0=vr0=vr0dc8meaabaqaciaacaGaaeqabaqabeGadaaakeaacuWG5bqEgaqcaaaa@2E37@ ← *doRelabel*(x, *θ*)

   5. until y^
 MathType@MTEF@5@5@+=feaafiart1ev1aaatCvAUfKttLearuWrP9MDH5MBPbIqV92AaeXatLxBI9gBaebbnrfifHhDYfgasaacH8akY=wiFfYdH8Gipec8Eeeu0xXdbba9frFj0=OqFfea0dXdd9vqai=hGuQ8kuc9pgc9s8qqaq=dirpe0xb9q8qiLsFr0=vr0=vr0dc8meaabaqaciaacaGaaeqabaqabeGadaaakeaacuWG5bqEgaqcaaaa@2E37@ remains constant

The *doSVM*() procedure can be any standard and complete implementation of an SVM classifier with support for nonlinear discriminator function. The idea is that *doSVM*() has to converge regardless of the geometry of the data, in order to provide the *doRelabel*() procedure with the hyperplane and other standard SVM outputs. After this procedure, *doRelabel*() reassigns some (or all) of the misclassified features to the other cluster. If D(x_i_, *θ*) is the distance between x_i _feature and the trained SVM hyperplane, then heavily misclassified feature, x_j ∈ J _could be selected by comparing D(x_j_, *θ*) to D(x_j'_, *θ*) for all j' ∈ J. Algorithm 2 clarifies the basic implementation of this procedure.

### Algorithm 2: doRelabel() Procedure

Require: Input vector: x

         Cluster labeling: y^
 MathType@MTEF@5@5@+=feaafiart1ev1aaatCvAUfKttLearuWrP9MDH5MBPbIqV92AaeXatLxBI9gBaebbnrfifHhDYfgasaacH8akY=wiFfYdH8Gipec8Eeeu0xXdbba9frFj0=OqFfea0dXdd9vqai=hGuQ8kuc9pgc9s8qqaq=dirpe0xb9q8qiLsFr0=vr0=vr0dc8meaabaqaciaacaGaaeqabaqabeGadaaakeaacuWG5bqEgaqcaaaa@2E37@

      SVM model: *θ*

      Confidence Factor: *α*Identify misclassified features:

            x'^+ ^← *K *misclassified features with y^
 MathType@MTEF@5@5@+=feaafiart1ev1aaatCvAUfKttLearuWrP9MDH5MBPbIqV92AaeXatLxBI9gBaebbnrfifHhDYfgasaacH8akY=wiFfYdH8Gipec8Eeeu0xXdbba9frFj0=OqFfea0dXdd9vqai=hGuQ8kuc9pgc9s8qqaq=dirpe0xb9q8qiLsFr0=vr0=vr0dc8meaabaqaciaacaGaaeqabaqabeGadaaakeaacuWG5bqEgaqcaaaa@2E37@ = +1

            x'^- ^← *L *misclassified features with y^
 MathType@MTEF@5@5@+=feaafiart1ev1aaatCvAUfKttLearuWrP9MDH5MBPbIqV92AaeXatLxBI9gBaebbnrfifHhDYfgasaacH8akY=wiFfYdH8Gipec8Eeeu0xXdbba9frFj0=OqFfea0dXdd9vqai=hGuQ8kuc9pgc9s8qqaq=dirpe0xb9q8qiLsFr0=vr0=vr0dc8meaabaqaciaacaGaaeqabaqabeGadaaakeaacuWG5bqEgaqcaaaa@2E37@ = -1

   1. for all i^th ^component of x'^+ ^do

   2. if i/K ∑^K^_j = 1 _D(x'^+^_j_, *θ*) <*α*D(x'^+^_j_, *θ*) then

   3. y^
 MathType@MTEF@5@5@+=feaafiart1ev1aaatCvAUfKttLearuWrP9MDH5MBPbIqV92AaeXatLxBI9gBaebbnrfifHhDYfgasaacH8akY=wiFfYdH8Gipec8Eeeu0xXdbba9frFj0=OqFfea0dXdd9vqai=hGuQ8kuc9pgc9s8qqaq=dirpe0xb9q8qiLsFr0=vr0=vr0dc8meaabaqaciaacaGaaeqabaqabeGadaaakeaacuWG5bqEgaqcaaaa@2E37@_*i*_^+ ^← -1

   4. end if

   5. end for

   6. for all ith component of x'^- ^do

   7. if 1/L ∑^L^_j = 1 _D(x'^-^_j_, *θ*) <*α*D(x'^-^_j_, *θ*) then

   8. y^
 MathType@MTEF@5@5@+=feaafiart1ev1aaatCvAUfKttLearuWrP9MDH5MBPbIqV92AaeXatLxBI9gBaebbnrfifHhDYfgasaacH8akY=wiFfYdH8Gipec8Eeeu0xXdbba9frFj0=OqFfea0dXdd9vqai=hGuQ8kuc9pgc9s8qqaq=dirpe0xb9q8qiLsFr0=vr0=vr0dc8meaabaqaciaacaGaaeqabaqabeGadaaakeaacuWG5bqEgaqcaaaa@2E37@_i_^- ^← +1

   9. end if

   10. end for

### SV-dropper

In most applications of clustering, the dataset is composed of *leverage *and *influential *points. *Leverage *points are subsets of the dataset that are highly deviated from the rest of the cluster, and removing them does *not *significantly change the result of the clustering. In contrast, *influential *points are those in the highly deviated subset whose inclusion or removal significantly changes the decision of the clustering algorithm. Effective, identification of these special points is of interest to improve accuracy and correctness of the clustering algorithm. A systematic way to manage these deviants is given by the SV-Dropper algorithm.

As depicted in Algorithm 3, SVM is initially trained on the clustered data; the weakest of the cluster data – those closest to the hyperplane, i.e., the support vectors – are dropped thereafter. This processed is repeated until the desired ratio of accuracy and number of data dropped is achieved.

### Algorithm 3: SV-Dropper Algorithm

Require: Input vector: x

   Cluster labeling: y^
 MathType@MTEF@5@5@+=feaafiart1ev1aaatCvAUfKttLearuWrP9MDH5MBPbIqV92AaeXatLxBI9gBaebbnrfifHhDYfgasaacH8akY=wiFfYdH8Gipec8Eeeu0xXdbba9frFj0=OqFfea0dXdd9vqai=hGuQ8kuc9pgc9s8qqaq=dirpe0xb9q8qiLsFr0=vr0=vr0dc8meaabaqaciaacaGaaeqabaqabeGadaaakeaacuWG5bqEgaqcaaaa@2E37@

1. let:

   x^+ ^← *K *features with *y *= +1

   x^- ^← *L *fatures with y^
 MathType@MTEF@5@5@+=feaafiart1ev1aaatCvAUfKttLearuWrP9MDH5MBPbIqV92AaeXatLxBI9gBaebbnrfifHhDYfgasaacH8akY=wiFfYdH8Gipec8Eeeu0xXdbba9frFj0=OqFfea0dXdd9vqai=hGuQ8kuc9pgc9s8qqaq=dirpe0xb9q8qiLsFr0=vr0=vr0dc8meaabaqaciaacaGaaeqabaqabeGadaaakeaacuWG5bqEgaqcaaaa@2E37@ = -1

2. repeat

3.    *θ *← *doSVM*(x, *y*)

4.    for all features, *x*_*j*_,

5. drop feature, *x*_*j*_, if *|*D(*x*_*j*_, *θ*)| < 1 end for

6. until desired ratio of SSE and number of data dropped

## Competing interests

The authors declare that they have no competing interests.

## Authors' contributions

The initial submission was written by SM and SWH, with revisions by SWH. The core SVM pattern recognition software and SVM-clustering method were developed by SWH. SM developed a separate SVM classifier for test validation and performed the SVM-clustering dataruns.
